# Consolidation therapy impact on survival outcomes in young patients with intracranial primary diffuse large B-cell lymphoma achieving complete remission: a propensity score matching analysis

**DOI:** 10.3389/fonc.2025.1493542

**Published:** 2025-03-31

**Authors:** Ge Wen, Miao Xiang, Hanyu Wang, Jijin Wang, Han Shao, Jinshan Zhang, Yujing Zhang

**Affiliations:** ^1^ Department of Radiation Oncology; Guangdong Provincial Key Laboratory of Major Obstetric Diseases; Guangdong Provincial Clinical Research Center for Obstetrics and Gynecology; The Third Affiliated Hospital, Guangzhou Medical University, Guangzhou, China; ^2^ Department of Radiation Oncology, State Key Laboratory of Oncology in South China, Guangdong Provincial Clinical Research Center for Cancer, Sun Yat-sen University Cancer Center, Guangzhou, China; ^3^ Department of Oncology, Mianyang Central Hospital, Mianyang, China

**Keywords:** diffuse large B-cell lymphoma, central nervous system neoplasm, radiotherapy, methotrexate, stem cell transplantation

## Abstract

**Objectives:**

Primary central nervous system lymphoma is an extremely aggressive type of non-Hodgkin lymphoma, and there is no consensus regarding the optimal management strategy for this disease. This study aimed to analyze the impact of consolidation therapy among young patients with intracranial primary diffuse large B-cell lymphoma (DLBCL).

**Methods:**

This retrospective study analyzed the clinical data of 55 young patients (age < 60 years) with intracranial primary DLBCL who achieved complete remission (CR) after high-dose methotrexate (HD-MTX)-based chemotherapy from March 2001 to October 2021. Among these patients, 33 patients received consolidation therapy, and 22 patients did not. Overall survival (OS) and disease-free survival (DFS) were compared between the two groups via Kaplan–Meier analysis, the multivariate Cox proportional hazards method, and propensity score matching (PSM).

**Results:**

The median follow-up time was 60.1 months. A total of 13 patients (23.6%) died, and 20 patients (36.4%) experienced recurrence. Patients who received consolidation therapy had higher 2-year OS (96.8% vs. 71.1%, *P* = 0.036) and DFS (90.9% vs. 56.4%, *P* = 0.006) rates than those without consolidation therapy. Multivariate analysis after PSM revealed that consolidation therapy was an independent predictor of DFS (HR = 0.282, 95% CI = 0.084–0.942, *P* = 0.040). Furthermore, rituximab was an independent predictor of favorable OS, and performance status was an independent predictor of OS and DFS. Subgroup analysis showed rituximab significantly improved OS in patients without consolidation therapy (88.9% vs. 45.0%, *P* = 0.006), but not in those with consolidation therapy (95.0% vs. 100%, *P* = 0.528).

**Conclusions:**

Consolidation therapy improved DFS in young intracranial primary DLBCL patients achieving CR after HD-MTX-based chemotherapy. Autologous stem cell transplantation and radiotherapy showed comparable consolidation benefits. Good performance status correlated with favorable outcomes. Adding rituximab to induction chemotherapy may improve OS in patients without consolidation therapy, but it might be unnecessary for those eligible for consolidation. Further research involving a larger patient cohort is warranted to ascertain rituximab’s efficacy.

## 1 Introduction

Primary central nervous system lymphoma (PCNSL) represents a highly aggressive subtype of non-Hodgkin lymphoma, predominantly manifesting as intracranial tumors, with a minority presenting as primarily intraocular or spinal cord lesions. PCNSL constitutes approximately 2% of all primary central nervous system tumors (1, 2). The most common type of PCNSL is diffuse large B-cell lymphoma (DLBCL), which has distinct biological properties and clinical behavior, resulting in a more aggressive nature than systemic DLBCL (3, 4). The introduction of high-dose methotrexate (HD-MTX) (dose ranging from 3.5 to 8.0 g/m²) as the standard systemic therapy has significantly enhanced treatment responses in PCNSL patients (5). However, a substantial subset of patients continues to exhibit resistance to upfront treatment, resulting in poor prognostic outcomes (6).

Following induction chemotherapy, the prevailing treatment strategies encompass consolidation therapy through whole-brain radiotherapy (WBRT) and high-dose chemotherapy in conjunction with autologous stem cell transplantation (ASCT) for patients who achieve complete remission (CR). In contrast, patients who do not attain CR are managed with salvage or palliative radiotherapy (7). Nonetheless, the effectiveness and safety of implementing consolidation therapy subsequent to HD-MTX induction therapy remain uncertain. Consequently, treatment regimens may vary considerably across institutions and regions.

This study aims to assess the impact of consolidation therapy on young patients with intracranial primary DLBCL by employing propensity score matching (PSM).

## 2 Methods

### 2.1 Patient selection and data collection

This retrospective study analyzed the clinical, treatment, and follow-up data of newly diagnosed PCNSL patients treated at the Sun Yat-sen University Cancer Center between March 2001 and October 2021. The patients were pathologically diagnosed in accordance with the fifth edition of the WHO Guidelines for the Classification of Tumors of the CNS (8).

The inclusion criteria were as follows: 1) age < 60 years, 2) primary lesion located in the brain, 3) pathologically confirmed DLBCL, and 4) CR was achieved after HD-MTX-based (dose ranging from 3.5 to 8.0 g/m^2^) chemotherapy according to the International PCNSL Collaborative Group 2005 criteria (9).

The exclusion criteria were as follows: 1) patients with solitary intraocular lymphoma, 2) patients with secondary PCNSL, 3) immunocompromised patients, 4) patients who underwent prior radiotherapy, 5) patients who were followed up for less than one month, 6) patients with other central nervous system diseases, and 7) patients with other malignant tumors.

Data from physical examinations, medical history, bone marrow biopsy, blood tests, and imaging were collected. The baseline characteristics included age, sex, lesion site, Eastern Cooperative Oncology Group performance status (ECOG PS), surgical approach, and immunohistochemistry.

### 2.2 Follow-up and statistical analysis

The recurrence and survival data of patients were obtained via regular outpatient re-examination or telephone follow-up, and census and surveillance data collected by government agencies were used for patients who were lost to follow-up.

A database was established via SPSS 26.0 (IBM Corp., Armonk, NY, USA). Figures were drawn via GraphPad Prism 9.0 (GraphPad Software, San Diego, CA, USA). Overall survival (OS) and disease-free survival (DFS) were the primary outcome measures and were examined via the Kaplan–Meier method with the log-rank test. The chi-square test was used to compare clinicopathological characteristics between the non-consolidation and consolidation therapy groups.

To balance the covariates between the non-consolidation and consolidation therapy groups, PSM analysis was conducted on confounding factors. Based on prior literature (10, 11), the inclusion criteria for the matching variables were as follows: 1) variables identified by univariate analysis as significantly associated (*P* < 0.10) with survival outcomes (ECOG PS, molecular subtype and targeted therapy); 2) variables that may potentially influence treatment selection and survival outcomes (sex, deep structure involvement and surgical procedure). A 1:1 ratio nearest neighbor match with a caliper value of 0.2 was used. A Cox proportional hazards model was used for multivariate analysis. All tests were two-tailed, and *P* < 0.05 was considered statistically significant.

## 3 Results

### 3.1 Patient characteristics

A total of 55 individuals were included in this study. All patients were < 60 years old at diagnosis, and the median age was 48 years (range, 15–60 years). Histopathological diagnoses were made in 15 patients via stereotactic biopsy, 18 patients via open biopsy and 22 patients via surgical resection. Following histological confirmation, all patients received HD-MTX-based (dose ranging from 3.5 to 8.0 g/m^2^) regimens with a median of 6 cycles (ranging from 2 to 10 cycles). Rituximab was administered to 36 patients. Specifically, the treatment regimens comprised HD-MTX–temozolomide–rituximab for 29 patients, HD-MTX–rituximab for 4 patients, and HD-MTX–cytarabine–rituximab for 3 patients.

All patients achieved CR after chemotherapy. Among them, 12 patients who had sufficient autologous peripheral blood stem cell collection proceeded to undergo ASCT as previously described (3, 12). The conditioning regimens consisted of carmustine and thiotepa for 7 patients, thiotepa, busulfan, and cyclophosphamide for 3 patients, and etoposide and thiotepa for 2 patients. A total of 21 patients received consolidation WBRT as previously defined (2). The median total dose was 40.0 Gy (ranging from 23.4 to 56.0 Gy), and the median whole-brain dose was 30.0 Gy (ranging from 20.0 to 40.0 Gy). In addition, 22 patients were monitored via a watchful waiting strategy. Among patients receiving rituximab during induction therapy, 10/36 (27.8%) underwent ASCT, 12/36 (33.3%) received WBRT, and 14/36 (38.9%) were managed with watchful waiting. For those not receiving rituximab, 2/19 (10.5%) underwent ASCT, 9/19 (47.4%) received WBRT, and 8/19 (42.1%) were managed with watchful waiting. **Table 1** summarizes the primary baseline characteristics of the participants.

**Table 1:** Univariate survival analysis based on baseline clinicopathologic characteristics for all patients (n = 55).

Characteristics	No. (%)	2-year OS (%)	*P* value	2-year DFS (%)	*P* value
All patients	55	87.7		78.7	
Sex			0.923		0.730
Male	29 (52.7)	84.4		77.7	
Female	26 (47.3)	91.3		80.0	
Age (years)			0.804		0.900
≤ 50	32 (58.2)	86.0		80.5	
> 50	23 (41.8)	90.3		76.4	
ECOG PS			0.002		0.002
0–1	38 (69.1)	94.3		89.4	
2–4	17 (30.9)	70.0		52.0	
LDH			0.706		0.291
Normal	46 (83.6)	85.2		74.5	
Elevated	9 (16.4)	100.0		100.0	
Deep structure involvement			0.429		0.793
No	23 (41.8)	86.1		77.1	
Yes	32 (58.2)	89.0		80.2	
Molecular subtype			0.081		0.080
Non-GCB	29 (52.7)	79.8		70.8	
GCB	26 (47.3)	96.0		87.8	
Surgical procedure			0.154		0.159
Resection	22 (40.0)	84.8		69.9	
Biopsy	33 (60.0)	89.7		84.5	
Rituximab			0.012		0.146
No	19 (34.6)	78.3		67.4	
Yes	36 (65.5)	93.1		85.2	
Consolidation therapy			0.036		0.006
No	22 (40.0)	71.1		56.4	
Yes	33 (60.0)	96.8		90.9	
Consolidation regimens			0.039		0.012
ASCT	12 (21.8)	100.0	Ref	91.7	Ref
WBRT	21 (38.2)	94.7	0.078	90.5	0.136
None	22 (40.0)	71.1	0.015	56.4	0.009

OS, overall survival; DFS, disease-free survival; ECOG PS, Eastern Cooperative Oncology Group performance status; LDH, lactate dehydrogenase; GCB, germinal center B-cell-like; ASCT, autologous stem cell transplantation; WBRT, whole-brain radiotherapy.

### 3.2 Survival outcomes

The date of the last follow-up was January 31, 2023, resulting in a median patient follow-up of 60.1 months (ranging from 3.6 to 118.1 months). Overall, 13 patients (23.6%) died, and 20 patients (36.4%) experienced disease recurrence. In the non-consolidation group, 7 patients (31.8%) died, and 11 patients (50.0%) experienced recurrence, while in the consolidation group, 6 patients (18.2%) died, and 9 patients (27.3%) experienced recurrence.

**Figure 1:**
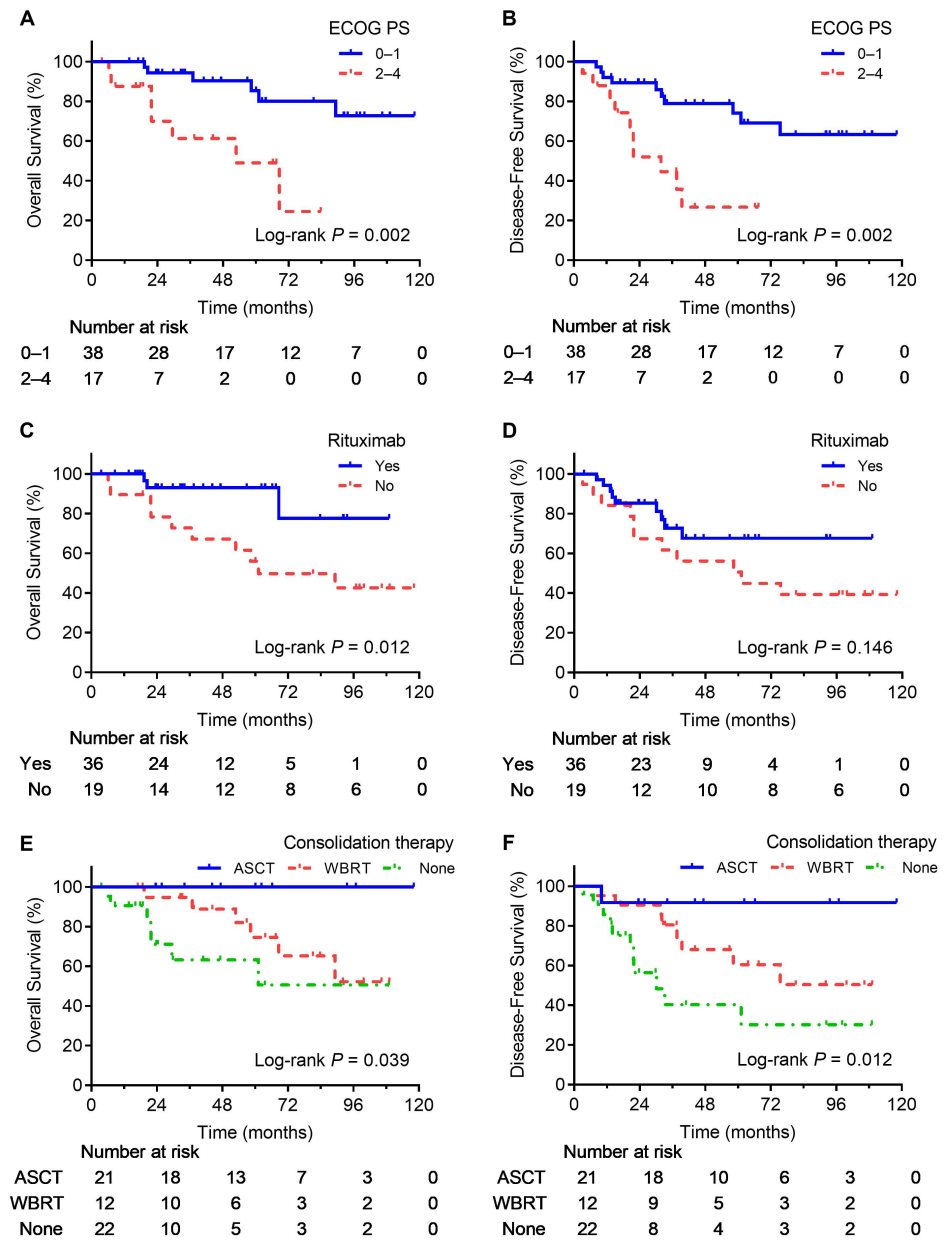
Survival curves for significant prognostic factors. OS **(A)** and DFS **(B)** curves for patients with different ECOG PS. OS **(C)** and DFS **(D)** curves for patients treated with or without rituximab. OS **(E)** and DFS **(F)** curves for patients treated with different consolidation regimens.

As shown in **Table 1**; **Figure 1**, survival outcomes were significantly better in patients receiving consolidation therapy than in the non-consolidation group, with no significant difference between WBRT and ASCT. Additionally, an ECOG PS of 0–1 was a predictor of favorable OS and DFS, and rituximab was a predictor of favorable OS. In addition, to further validate the effect of rituximab, we performed a stratified survival analysis of rituximab based on whether patients received consolidation therapy. The results indicated that rituximab significantly improved OS in patients who did not receive consolidation therapy, whereas no significant differences in DFS and OS were observed among patients who received consolidation therapy (**Table 2**).

**Table 2:** Stratified survival analysis of rituximab by consolidation therapy for all patients (n = 55).

Groups	No.	2-year OS (%)		*P* value	2-year DFS (%)		*P* value
		Non-rituximab (n)	Rituximab (n)		Non-rituximab (n)	Rituximab (n)	
Consolidation							
No	22	45.0 (8)	88.9 (14)	0.006	30.0 (8)	74.1 (14)	0.207
Yes	33	100 (11)	95.0 (22)	0.528	90.9 (11)	90.9 (22)	0.487

OS, overall survival; DFS, disease-free survival.

### 3.3 Patient characteristics before and after PSM

Before PSM, the proportion of patients who underwent biopsy was greater in the consolidation group. PSM analysis was performed in the non-consolidation and consolidation groups based on sex, ECOG PS, deep structure involvement, molecular subtype, surgical procedure, and targeted therapy. A total of 20 pairs of patients were included, and the patient characteristics of the two groups were balanced after PSM (**Table 3**).

**Table 3:** Distribution of characteristics in the non-consolidation and consolidation groups before and after PSM.

Characteristics	Before PSM		χ²	*P* value	After PSM		χ²	*P* value
	Non- consolidation (n = 22) %	Consolidation (n = 33) %			Non- consolidation (n = 20) %	Consolidation (n = 20) %		
Sex			0.05	0.825			0.00	1.000
Male	12 (54.6)	17 (51.5)			10 (50.0)	10 (50.0)		
Female	10 (45.5)	16 (48.5)			10 (50.0)	10 (50.0)		
Age (years)			1.01	0.315			1.62	0.204
≤ 50	11 (50.0)	21 (63.6)			9 (45.0)	13 (65.0)		
> 50	11 (50.0)	12 (36.4)			11 (55.0)	7 (35.0)		
ECOG PS			1.72	0.190			0.48	0.490
0–1	13 (59.1)	25 (75.8)			13 (65.0)	15 (75.0)		
2–4	9 (40.9)	8 (24.2)			7 (35.0)	5 (25.0)		
LDH			0.00	1.000			0.20	0.658
Normal	18 (81.8)	28 (84.9)			16 (80.0)	18 (90.0)		
Elevated	4 (18.2)	5 (15.2)			4 (20.0)	2 (10.0)		
Deep structure involvement			0.20	0.655			0.40	0.525
No	10 (45.5)	13 (39.4)			10 (50.0)	8 (40.0)		
Yes	12 (54.6)	20 (60.6)			10 (50.0)	12 (60.0)		
Molecular subtype			0.60	0.440			0.11	0.744
Non-GCB	13 (59.1)	16 (48.5)			13 (65.0)	12 (60.0)		
GCB	9 (40.9)	17 (51.5)			7 (35.0)	8 (40.0)		
Surgical procedure			5.57	0.018			1.62	0.204
Resection	13 (59.1)	9 (27.3)			11 (55.0)	7 (35.0)		
Biopsy	9 (40.9)	24 (72.7)			9 (45.0)	13 (65.0)		
Rituximab			0.05	0.817			0.00	1.000
No	8 (36.4)	11 (33.3)			7 (35.0)	7 (35.0)		
Yes	14 (63.6)	22 (66.7)			13 (65.0)	13 (65.0)		

PSM, propensity score matching; ECOG PS, Eastern Cooperative Oncology Group performance status; LDH, lactate dehydrogenase; GCB, germinal center B-cell-like.

### 3.4 Multivariate analysis

**Figure 2:**
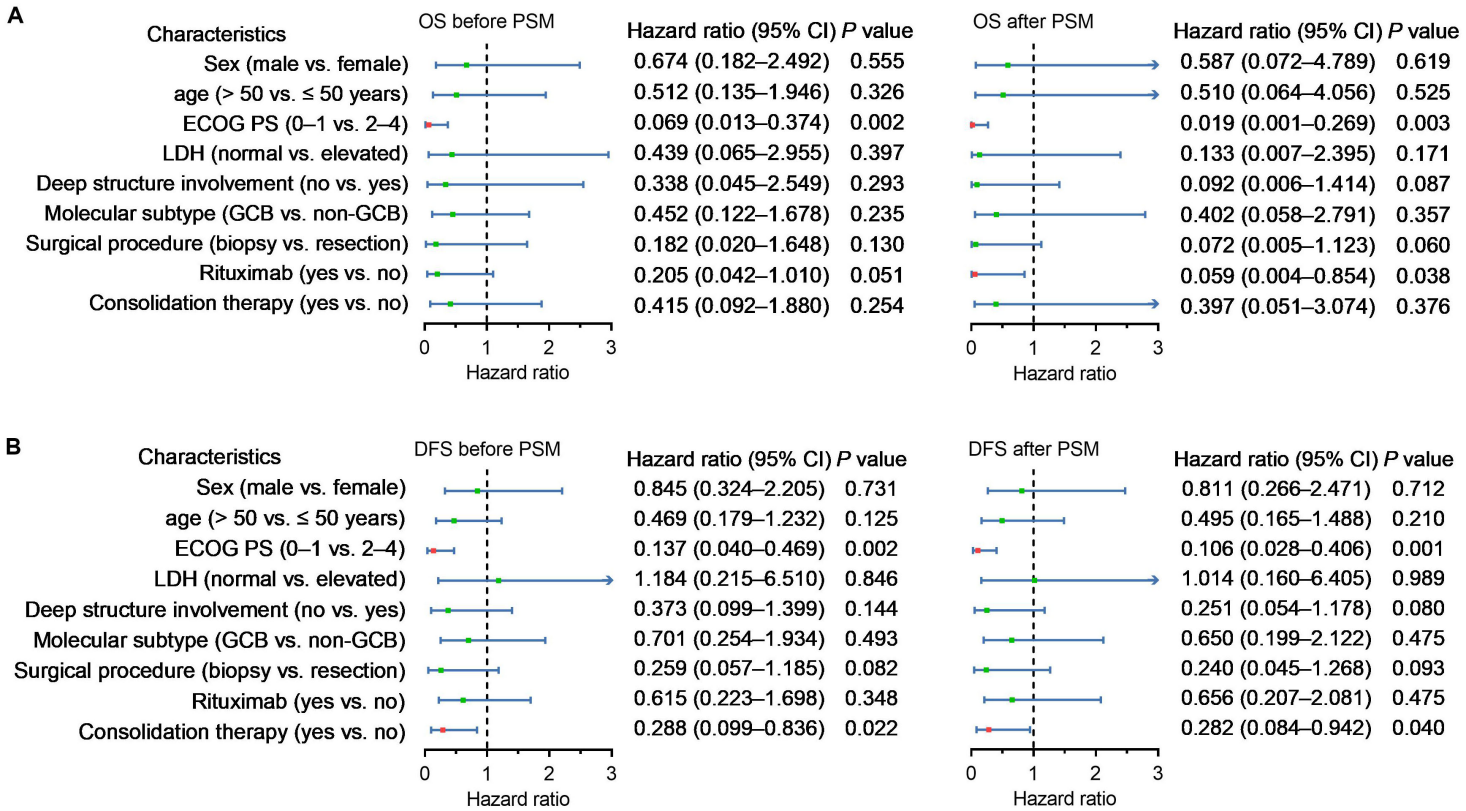
Forest plots representing multivariate analysis of risk factors associated with OS **(A)** and DFS **(B)** for all patients and matched patients. The bars represent the 95% confidence intervals of the hazard ratios.

Before PSM, the ECOG PS was an independent predictor for OS and DFS, and consolidation therapy was an independent predictor for DFS. After PSM, rituximab-based therapy was also found to be an independent predictor of OS in addition to the above findings (**Figure 2**).

### 3.5 Neurotoxicity

Treatment-related neurotoxicity was defined as progressive neurological or cognitive impairment confirmed by serial clinical examinations in the absence of recurrent lymphoma. A total of 5 patients (23.8%) who received consolidation WBRT showed clinical evidence of treatment-related neurotoxicity, which included leukoencephalopathy in 2 patients, memory impairment in 2 patients, and cognitive disturbance in 1 patient. Among patients receiving ASCT as consolidation therapy, no patients developed treatment-related neurotoxicity.

## 4 Discussion

HD-MTX-based chemotherapy is currently endorsed as the induction therapy for patients newly diagnosed with PCNSL (3, 7). A meta-analysis of clinical trials has demonstrated that HD-MTX-based regimens achieve an overall CR rate of 41% in this patient population (13). Consequently, we included patients undergoing HD-MTX-based induction chemotherapy in the present study.

While the addition of rituximab has been shown to enhance the prognosis of patients with systemic DLBCL, clinical investigations into its efficacy in PCNSL patients have produced inconsistent findings. For instance, a randomized phase III clinical trial (HOVON 105/ALLG NHL 24) indicated that the incorporation of rituximab into HD-MTX-based induction chemotherapy did not lead to improved disease outcomes (14, 15). In contrast, a phase II trial conducted by the International Extranodal Lymphoma Study Group-32 (IELSG 32) reported that the combination of HD-MTX–cytarabine with rituximab significantly increased the CR rate (30% vs. 23%), 7-year PFS rate (29% vs. 20%), and OS rate (37% vs. 21%) (5, 16). The divergence in outcomes between these two trials may be attributed to variations in protocol design and treatment intensity. A meta-analysis of two randomized trials indicated that the combination of rituximab with HD-MTX-based induction chemotherapy prolonged PFS, although its effects on OS remain uncertain (17). Another meta-analysis examining the impact of rituximab in patients with PCNSL found that rituximab use was significantly associated with higher CR rates and improved OS and PFS at both 3 and 5 years. This suggests that rituximab may positively influence the prognosis of PCNSL patients (18). Our findings revealed that, while DFS did not exhibit a statistically significant enhancement, patients undergoing rituximab combination therapy experienced an improvement in OS. This observation may be attributed to the proposed interaction between WBRT and rituximab, wherein WBRT-induced disruption of the blood-brain barrier potentially facilitates increased brain penetration of residual rituximab, thereby contributing to the observed OS improvement (15). Additionally, the effect of consolidation therapy on DFS may have also impacted the DFS survival analysis in the context of rituximab treatment. Notably, subgroup analysis stratified by consolidation therapy revealed that the OS benefit of rituximab was noticed in patients without consolidation therapy (88.9% vs. 45.0%, *P* = 0.006), but not in those with consolidation therapy (95.0% vs. 100%, *P* = 0.528). This finding, along with the PSM-adjusted multivariate analysis, suggested that rituximab contributes to OS improvement independently of consolidation therapy, which implied that the inclusion of rituximab in induction regimens may not provide additional survival benefits for patients undergoing consolidation therapy. Therefore, rituximab may be considered unnecessary in the induction therapy for young PCNSL patients eligible for consolidation strategies. Considering the limited sample size of the present study, further validation in a larger patient cohort is warranted to ascertain the efficacy of rituximab in young patients with PCNSL. A *post-hoc* analysis of the Phase III HOVON 105/ALLG NHL 24 trial demonstrated that rituximab significantly enhanced event-free survival in patients aged 60 years or younger who received WBRT. Nevertheless, the authors urge caution in interpreting these findings and recommend a randomized Phase III study specifically targeting OS in patients under 60 with PCNSL to validate rituximab’s efficacy (15). Our results provide retrospective support for this recommendation.

As a consolidation to HD-MTX chemotherapy, WBRT has been shown to extend survival in patients with PCNSL (19, 20). However, the effectiveness of consolidation WBRT remains contentious. A Phase III randomized trial conducted by the German PCNSL Study Group indicated that the addition of WBRT to the HD-MTX regimen prolonged PFS but did not enhance OS and increased neurotoxicity compared to HD-MTX-based monotherapy (21). Gavrilovic et al. reported that WBRT following the HD-MTX regimen significantly extended OS in patients under 60 years, with 74% surviving until the final follow-up. They recommended deferring WBRT in patients over 60 to mitigate treatment-related neurotoxicity, as the median OS was 29 months, irrespective of WBRT administration (22). Furthermore, existing literature suggests that consolidation WBRT does not enhance survival rates in patients who achieve CR following HD-MTX-based induction chemotherapy, indicating that the omission of WBRT could be considered for these patients (23). Contrarily, research by Omuro et al. indicates that for PCNSL patients under 60 years of age who achieve CR after HD-MTX induction chemotherapy, deferring WBRT may not be optimal due to suboptimal PFS (24). In our study, multivariate analysis following PSM demonstrated a significant improvement in DFS but not OS in patients who achieved CR after HD-MTX induction chemotherapy and received consolidation therapy, including WBRT and ASCT, compared to those who did not receive consolidation therapy.

The primary concern associated with WBRT in patients with PCNSL is the risk of delayed neurotoxicity, which undermines the long-term survival advantages, particularly among elderly individuals (20, 23). ASCT has emerged as an effective and promising alternative consolidation strategy to WBRT following HD-MTX-based induction therapy. Evidence from two randomized phase II trials, IELSG 32 and PRECIS, indicates that ASCT as a consolidation therapy is noninferior to WBRT in terms of PFS and OS for patients newly diagnosed with PCNSL (16, 25). Furthermore, both studies revealed that ASCT is significantly associated with the preservation of neurocognitive function. However, it is important to note that treatment-related mortality and hematologic toxicity rates were significantly higher with ASCT compared to WBRT (16, 25). A meta-analysis of PCNSL consolidation therapies found no significant differences between ASCT and WBRT in terms of OS and PFS. Nonetheless, concerning neurocognitive function, patients receiving WBRT exhibited a significant decline in attention and executive function (26). Our study produced comparable results, indicating no significant difference in survival outcomes between patients undergoing WBRT and those undergoing ASCT following CR with HD-MTX-based chemotherapy. Additionally, treatment-related neurotoxicity was observed in patients who received WBRT, whereas those who underwent ASCT did not experience significant neurocognitive decline. This finding suggests that ASCT may serve as a viable alternative to reduce WBRT-associated neurotoxicity in younger patients who prioritize long-term cognitive preservation. Therefore, when selecting a consolidation therapy regimen, it is crucial to thoroughly consider patients’ age, performance status, and the potential impact on their quality of life (26). In this context, ASCT may be more suitable for younger patients who respond favorably to induction chemotherapy (12).

Among the predictors of PCNSL, the ECOG PS has been identified as a key baseline predictor (27). In this study, patients with a favorable ECOG PS demonstrated significantly better survival outcomes.

This study is subject to several potential limitations. As a retrospective analysis, our assessment of the neurotoxicity profile was constrained to clinical records, rather than formal neuropsychological testing. Additionally, the findings may be affected by selection bias and variability in management practices. Nevertheless, the study’s focus on data from patients treated at a single hospital allowed for the standardization and consistency of diagnostic methods, treatment protocols, and follow-up procedures. Moreover, we utilized PSM to mitigate the influence of confounding variables, thereby enhancing the robustness of the evidence supporting the efficacy of consolidation therapy.

This study concentrated on patients under 60 years of age with intracranial primary DLBCL who attained CR following HD-MTX induction chemotherapy. Our results indicate that consolidation therapy significantly enhances DFS in this cohort, thereby informing clinical decision-making regarding consolidation strategies. Furthermore, our research demonstrated comparable efficacy between WBRT and ASCT as consolidation therapies concerning survival outcomes. This finding offers clinicians alternative treatment options, particularly considering the potential neurotoxic effects associated with WBRT. ASCT may be recommended for younger patients who prioritize the preservation of long-term cognitive function. Lastly, the observed potential benefit of rituximab in improving OS further supports its inclusion in induction chemotherapy regimens.

## 5 Conclusions

In young patients diagnosed with intracranial primary DLBCL who achieved CR following HD-MTX-based chemotherapy, those who underwent consolidation therapy exhibited superior DFS outcomes. The efficacy of consolidation therapy was comparable between ASCT and WBRT. Patients exhibiting a favorable ECOG PS also experienced improved prognoses. Additionally, the incorporation of rituximab into the HD-MTX-based chemotherapy regimen was associated with improved OS in patients who did not receive consolidation therapy. Conversely, rituximab may be considered unnecessary for induction therapy in patients eligible for consolidation strategies. Further investigation is warranted to ascertain the efficacy of rituximab within a larger patient cohort.

## Data Availability

The raw data supporting the conclusions of this article will be made available by the authors, without undue reservation.
